# Synthesis of Vertically Oriented Graphene Sheets or Carbon Nanowalls—Review and Challenges

**DOI:** 10.3390/ma12182968

**Published:** 2019-09-12

**Authors:** Alenka Vesel, Rok Zaplotnik, Gregor Primc, Miran Mozetič

**Affiliations:** Department of Surface Engineering, Jozef Stefan Institute, Jamova cesta 39, 1000 Ljubljana, Slovenia; alenka.vesel@guest.arnes.si (A.V.); rok.zaplotnik@ijs.si (R.Z.); gregor.primc@ijs.si (G.P.)

**Keywords:** carbon nanowalls, plasma synthesis, growth mechanism, deposition speed, deposition parameters, deposition temperature

## Abstract

The paper presents a review on the current methods for deposition of vertically oriented multilayer graphene sheets (often called carbon nanowalls—CNWs) on solid substrates. Thin films of CNWs are among the most promising materials for future applications in capacitors, batteries, electrochemical devices, and photovoltaics, but their application is currently limited by slow deposition rates and difficulties in providing materials of a desired structure and morphology. The review paper analyzes results obtained by various groups and draws correlations between the reported experimental conditions and obtained results. Challenges in this scientific field are presented and technological problems stressed. The key scientific challenge is providing the growth rate as well as morphological and structural properties of CNWs thin films versus plasma parameters, in particular versus the fluxes of reactive plasma species onto the substrate surface. The technological challenge is upgrading of deposition techniques to large surfaces and fast deposition rates, and development of a system for deposition of CNWs in the continuous mode.

## 1. Introduction

**Figure 1:**
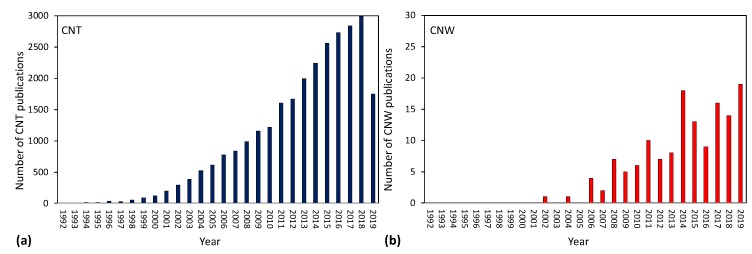
Number of publications per year regarding carbon nanotube (CNT) synthesis (**a**) and carbon nanowall (CNW) synthesis (**b**). Source: Web of Science.

Nanocarbon has attracted enormous attention in the past two decades. It can exist in various configurations such as graphene, carbon nanotubes (CNTs), or carbon nanowalls (CNWs). Robert F. Curl Jr., Sir Harold W. Kroto and Richard E. Smalley received the Noble prize in chemistry in 1996 for discovery of fullerenes, while Andre Geim and Konstantin Novoselov in physics in 2010 for ground-breaking experiments regarding the two-dimensional material graphene. S. IIjima has been a candidate for the prize, too, for discovery of carbon nanotubes, and he received the first Kavli prize in Nanotechnology in 2008. Many research groups are nowadays involved in research on nanocarbon worldwide. In Figure 1, a comparison of publications per year on a synthesis of CNTs and CNWs is shown. Nanocarbon in the form of CNWs has attracted less attention but represents a promising material for application in fuel cells, lithium ion batteries, photovoltaic devices, thin-film transistors, sensors of specific gaseous molecules, field-emission devices, batteries, light absorbers, enhanced detectors for electrochemical and gas sensors, supercapacitors and scaffolds for tissue engineering [1,2,3,4,5,6,7,8]. The unique property of carbon nanowalls versus any other known material is a combination of stability, chemical inertness, electrical conductivity, and huge surface-to-mass ratio. Carbon nanowalls are often referred to as “multilayer graphene sheets stretching perpendicularly to the substrate surface”. Such vertically oriented graphene sheets have a high density of atomic-scale graphitic edges that are potential sites for electron field emission [9]. Due to their high surface-to-mass ratio they are also good candidates for biosensors and energy storage applications [9]. Since awarding the Nobel prize, tens of thousands research groups have been involved in basic research as well as in application of graphene worldwide and promising results were reported; however, mass application of this type of carbon is yet to be realized. A way to implement this material in a mass production is depositing graphene perpendicular to a substrate surface and thus taking full advantage of its unique properties. This review paper intends to present current state-of-the-art on methods for deposition of carbon nanowalls as well as their properties where they are reported. Exact growth mechanisms are far from being well-understood; therefore, both theoretical and experimental study is yet to be performed. 

**Figure 2:**
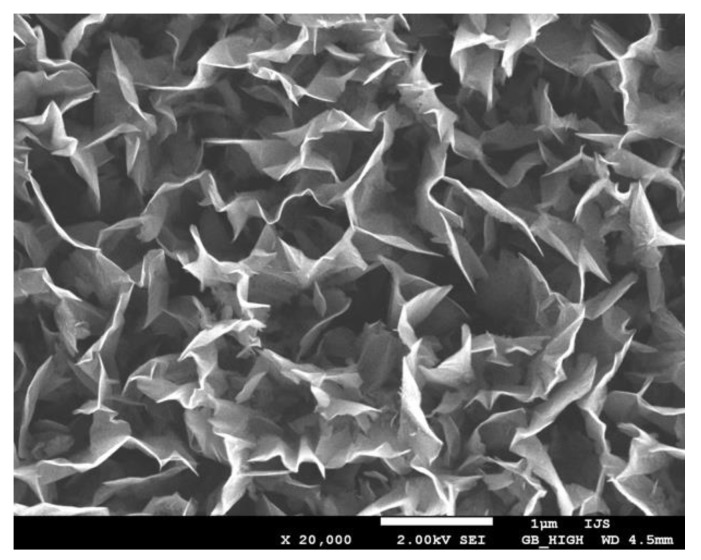
An example of carbon nanowalls grown on the surface of a titanium foil.

**Figure 3:**
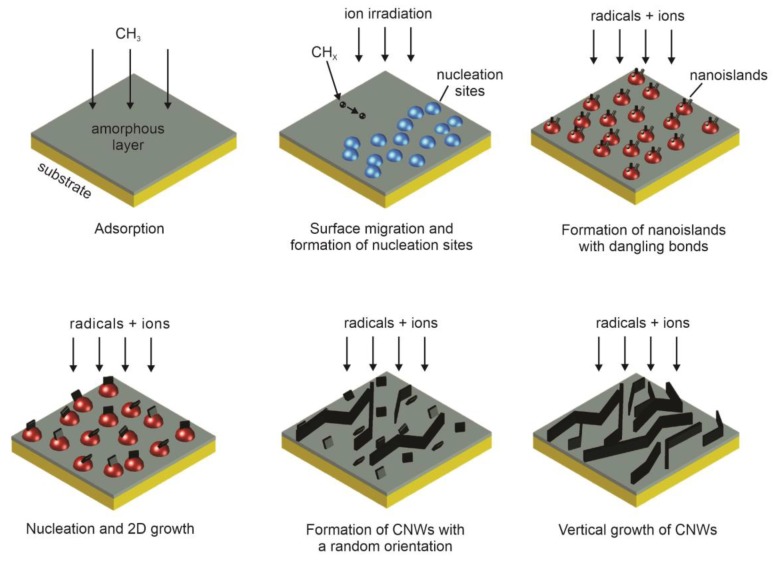
Schematic presentation of CNWs growth mechanism as suggested by Hiramatsu [9].

A typical SEM image of CNWs is shown in Figure 2. CNWs were first synthesized more than 10 years ago and can be deposited onto a substrate using a classical plasma-enhanced chemical vapor deposition (PECVD) method [10,11,12,13]. A carbon-containing gas (usually methane CH_4_ or acetylene C_2_H_2_) is partially atomized and ionized upon plasma conditions and the resultant radicals condensate on a substrate surface. Upon limited range of experimental conditions, carbon in the form of nanowalls (multilayer graphene sheets) grows on the substrate surface. The commonly accepted growth mechanism for CNWs is illustrated in Figure 3 and can be summarized as follows [9,14]:

1. Adsorption of CH_3_ radicals and formation of amorphous carbon layer on the substrate.
2. Formation of defects and dangling bonds because of ion irradiation leading to the formation of nucleation sites.
3. Migration of carbon species and formation of nanoislands with dangling bonds.
4. Nucleation of small graphene nanosheets on dangling bonds followed by a two-dimensional growth.
5. Formation of nanographene sheets with a random orientation.
6. Bonding of reactive carbon species to the edge of graphene sheets. Nanosheets that are standing almost vertically preferably grow faster and shadow low-lying graphene sheets, therefore their growth is suppressed.

Images taken by scanning electron microscopy (SEM) at various deposition times confirm the above mechanism, at least for experimental conditions adopted by [13,15,16].

Hydrogen was found to play a significant role in the growth process of CNWs by numerous authors [17,18,19]. Hydrogen is needed to etch and remove any amorphous carbon that is formed on the substrates, furthermore, it prevents formation of additional graphene layers by etching weakly bounded carbon atoms and it was found to enhance migration of carbon precursors [14]. Therefore, addition of hydrogen can greatly improve the quality of CNWs. The quality of CNWs can be also enhanced by addition of argon and even oxygen. By addition of argon and/or oxygen it was found to be possible to control the density of nanoislands in the initial stage of growth and thus consequently also the density of CNWs. This is the reason why CNWs are most frequently synthesized using a mixture of CH_4_ with H_2_ and Ar, where researchers usually use different pressures and gas flows to influence the deposition rate as well as quality of CNWs.

CNWs can be also formed by employing C_2_F_6_ gas in a mixture with hydrogen or oxygen [16]. In this case, C and CF_3_ radicals serve as building blocks, whereas hydrogen atoms are needed for abstraction of fluorine from the growing film. A mechanism for CNWs growth in a non-equilibrium C_2_F_6_ gas environment was proposed by Kondo et al. [16]. Another example of CNWs synthesis is an application of a CO/H_2_ gas environment upon heavily non-equilibrium conditions [20]. CNWs were observed only if H_2_ was added, whereas formation of nanofibers was observed if CO was mixed with Ar/O_2_ [21]. In recent years, CNWs were also successfully synthesized by using other precursors like organic precursor (p-xylene [22], ethanol or hexane [23,24]) or metal-organic precursors (aluminum acetylacetonate [25]).

A drawback of the currently known techniques for synthesizing CNWs is a low growing rate and inability to obtain uniform coatings on large substrates. Therefore, they are inappropriate for industrial application at this stage of the scientific knowledge. So far, researchers have managed to obtain uniform coatings on surfaces measured in square centimeters and the growth is accomplished in a time scale of minutes if not hours. The key problem arises from the fact that deposition rates using CH_x_ radicals cannot be enhanced because carbon agglomerates at elevated pressure (forming dusty plasma); therefore, the structure of the deposit is not appropriate—instead of nanowalls, carbon of various morphological shapes including soot or hydrogenated amorphous carbon grows at elevated pressure. So far, few alternatives to CH_x_ radicals have been reported except for CO and C_2_F_6_ as mentioned above.

Another problem limiting the application of carbon nanowalls on industrial scale is associated with deposition of CNWs at rather high substrate temperatures. Currently available methods allow deposition of CNWs only at temperatures in the range of 600–800 °C that are not appropriate for deposition of CNWs on polymer substrates [10]. Temperatures even higher than 800 °C were also reported. The researchers observed that the quality of CNWs is increasing with increasing temperature and also their growth rate [17,19,25,26,27,28,29]. Substrate temperature has therefore an important effect on their size and density. If the temperature was too low, CNWs did not grow and only an amorphous carbon layer was observed, or carbon in other morphological nanostructures [26,29]. Tii et al. found that addition of Ar to N_2_/CH_4_ gas system could lower the substrate temperature for CNWs deposition to approximately 650 °C [30]. Contrary, Park et al. reported deposition of CNWs at temperatures a bit lower than 400 °C (depending on the substrate material) [31]. Additionally, Singh [32] managed to synthesize CNWs on a glass surface at a temperature of approximately 400 °C, however he used hot-wire chemical vapor deposition. Although it was reported that graphene films have been deposited at low temperatures such as 240 °C [33], and carbon nanotubes (CNT) at just 120 °C [34], it is still a big challenge for researchers working on deposition of vertically oriented graphene sheets to minimize the substrate temperature. In one experiment, CNWs were deposited onto SiO_2_ substrates at various substrate temperatures ranging from 600 to 800 °C [26]. The deposition was performed using ECR-PECVD (electron-cyclotron resonance plasma-enhanced chemical vapor deposition) in CH_4_/Ar environment. The authors found an important effect of the substrate temperature on the vertical growth of CNWs through nanoscale graphitic islands. In this paper, 600 °C was found to be a minimum temperature where formation of nano-graphitic islands was observed. These two-dimensional nanoislands changed to three-dimensional structures when the substrate temperature was increased to 625 °C. However, further increase of the sample temperature led to formation of a higher density of CNWs. Their height and growth rate increased with increasing temperature and a formation of nest-like structure was observed [26]. In another experiment, Gentoiu et al. [29] found strong dependence of a structure, morphology, and graphitization on deposition temperature. At low temperatures ~200 °C carbon nanotubes (CNTs) were formed, at temperatures ~300–400 °C rather amorphous carbon nanoparticles appeared, whereas at temperatures ~500–700 °C formation of CNWs was observed. These experiments clearly show that the temperature has an important effect on the morphology and growth of CNWs. Deposition of CNWs is thus currently still limited to materials that can withstand high temperatures. 

Besides temperature, also the choice of a substrate material may influence the initial stage of the carbon cluster nucleation because of a different lattice matching with graphite and consequently different quality of CNWs may be formed [10]. In addition, carbon solubility in the substrate material may have a strong influence on the nucleation and growth of CNWs as reported by Giese at al. [25]. The authors investigated deposition of CNWs on various substrates including stainless steel, aluminum, nickel and silicon which strongly differ in carbon solubility. The effect of a bias voltage and substrate temperature was investigated as well. With increasing bias voltage and temperature, the morphology changed from nanorods which were formed at low bias voltage and temperature, to thorny structures, followed by straight CNWs, whereas curled CNWs were formed at high bias voltages and temperatures. These growth regimes were shifted for different materials. On stainless steel and aluminum all mentioned structures were formed, however they appeared at different bias voltage and temperature. Whereas for Ni and Si, no nanorods were formed and only straight and curled CNWs were found. This was explained by difference in carbon bulk and surface diffusion for these materials and different affinity to form carbides at the surface. Additionally, Vizireanu et al. [35] investigated the effect of substrate material on CNWs synthesis and their morphology. CNWs were deposited on various substrates including SiO_2_/Si, titanium, stainless steel, Quartz, MgO, and carbon paper, that were previously covered with clustered nickel catalyst. The authors found that the type, morphology, and electrical characteristics (conductive, insulator, or semiconductor) were not important for CNWs growth.

## 2. Early Scientific Documents of Plasma Synthesis of Carbon Nanowalls

An excellent and comprehensive review summarizing earlier achievements in CNWs deposition was prepared by Himatsu and Hori in 2010 [14]. Appropriate references of the earlier papers are summarized in this classical monograph. The first report on the synthesis of CNWs structures appeared in scientific literature in 2002 by Wu et al. [36]. The gases used were methane and hydrogen of flow rates 40 and 10 sccm, respectively. Such a gas mixture is a natural choice for depositing any carbon nanomaterials, because methane partially dissociates and ionizes upon plasma conditions and the radicals such as C, CH, CH_2_, and CH_3_ stick to the substrate surface. A rather high substrate temperature of about 700 °C was used to favor decomposition of hydrogenated carbon radicals to almost pure carbon suitable for growing carbon structures almost free from hydrogen. Additional DC biasing was applied to deliver more energy to the substrates upon growing of CNWs. A catalyst (typically NiFe) was applied to stimulate the nucleation. Addition of hydrogen was essential because atomic hydrogen and positively charged ions caused removal of weaker-bonded carbon what was found beneficial for appropriate structure of the CNWs. Wu et al. found their CNWs suitable for application in batteries, light-emitting and conversion devices, catalysts, and other areas requiring high surface area materials. 

In 2005 the group of Shiji [37] reported fabrication of CNWs by capacitively coupled radio-frequency plasma enhanced chemical vapor deposition (CCP-PECVD) employing fluorocarbon/hydrogen mixtures. Correlation between CNWs growth and fabrication conditions, such as the carbon source gases, was investigated. In addition, the influence of H-atom density in the plasma was measured using vacuum ultraviolet absorption spectroscopy to discuss the growth mechanism of CNWs. 

Additionally, also in 2005, Tanaka et al. [38] reported growth of CNWs on a SiO_2_ substrate by microwave plasma enhanced chemical vapor deposition. They investigated the growth process and revealed that the CNWs grew at the fine-textured structure on SiO_2_ and the growth process did not require the catalyst (as opposite to Wu et al.). It was found that the height of CNWs as a function of growing time obeyed the square-root law. Rather high growth rates of approximately 10 micrometers per hour were achieved. They also used hydrocarbons with hydrogen as a useful gas mixture.

Dikonimos et al. [39] reported CNWs with a maximum longitudinal dimension ranging from 10 to 200 nm and a wall thickness lower than 5 nm. Such structures were grown in a high-frequency chemical vapor deposition reactor on Si substrates. The growth precursor was methane diluted with a noble gas (He). The growth rate and film morphology were explored. The experimental setup consisted of a two-grid system which allowed to vary the voltage and current density on the substrate surface independently. An increase of growth rate was observed as the film thickness increased from a few nanometers to about 200 nm when the substrate current density was increased. 

The importance of hydrogen in the gas mixtures was elaborated by Cui et al. [17]. Without addition of H_2_, graphite sheets were difficult to produce, and the film contained other forms of carbon. At small H_2_ fluxes (40 sccm), the carbon nano-sheets were not clearly distinguished. When H_2_ flux was increased the vertical graphene sheets became more obvious (80–120 sccm). However, if the H_2_ flow was too high (150 sccm) the density of the vertical sheets decreased. At low H_2_ flow rates, the supply of hydrogen was insufficient to etch away the amorphous part, however at high H_2_ flow rates also CNWs were etched by excessive hydrogen [17]. Therefore, at optimal conditions a mixture should contain just the right density ratio of CH_x_ radicals acting as a source of carbon species and hydrogen atoms needed to etch away the amorphous part. Similar findings were also found by Jiang et al. [18].

Teii et al. [30] revealed the importance of Ar in the production of C_2_ dimers, which were found to be the most important radicals responsible for CNWs growth. He performed synthesis of CNWs in ASTex microwave plasma using Ar/N_2_/CH_4_ or Ar/N_2_/C_2_H_2_ gas mixtures with various Ar concentrations. The amount of C_2_ dimers was increasing by adding Ar. Furthermore, addition of Ar reduced the substrate temperature needed for CNWs deposition to 650 °C. Rather high deposition rates of approximately 1 µm/min were obtained.

Vizireanu et al. [35] synthesized CNWs structures in Ar/H_2_/C_2_H_2_ mixture on various substrates including SiO_2_/Si, Ti, stainless steel, Quartz glass, MgO, carbon paper, that were previously covered with clustered Ni catalyst. SEM images of CNWs on various substrates revealed that the type, morphology and electrical characteristics of the substrates (conductive, insulator, or semiconductor) were not important for CNWs growth. A deposition rate was approximately 1 µm per 30 min. The authors also investigated the influence of the pressure and gas flows. It was found that the quality of CNWs could be altered by changing the pressure or Ar flow. CNWs with large length-to-thickness ratio and well-isolated between themselves were deposited at low pressure and high carrier flow rates, whereas poor quality of CNWs was obtained at high pressure or low Ar flow.

Jiang et al. [18] also investigated the morphology of CNWs grown in CH_4_/H_2_ mixture at various CH_4_ flow rates and CH_4_ to H_2_ ratios. CH_4_ flow rate was changed from 5 to 100 sccm whereas H_2_ flow rate was kept constant. It was found that the size of graphene sheets first increased with increasing CH_4_ flow rate, reached a maximum in the range of flows 10–30 sccm, and then decreased with further increase of the CH_4_ flow rate. This result was explained by higher density of nucleation sites, faster nucleation, and sufficient density of carbon radicals with increasing flow rate. However, if the flow rate was too high, too high density of the nucleation sites was reported, thus hindering the nucleus from growing into large sizes of graphene sheets because of insufficient interspace between the neighbouring nuclei. Moreover, when CH_4_ flow rate was manipulated, also CH_4_ to H_2_ ration was changed which influenced the etching effect of hydrogen radicals. Too high H_2_ content led to a small size of the graphene sheets because of the excessive etching according to Jiang. Therefore, it was concluded that controlling the dynamic competition between growth and etching was the key factor for obtaining good quality of CNWs.

Davami et al. [40] investigated the morphology of CNWs grown in CH_4_/H_2_ systems on various substrates including Cu, Si, or Si coated with a thin layer of Ni or Au. The authors found that CNWs on pure Si substrates were denser and thinner in comparison to CNWs deposited on Si/Ni or Si/Au substrates, whereas CNWs on Cu were much finer than on all other substrates. 

The growth rate of PECVD techniques is usually limited to tens of nanometers per minute that is insufficient for practical applications. Zhang et al. [41] used “high density meso-plasma CVD” and obtained fast growth rate of the order of ~10 µm/min, depending on a power of a radio-frequency (RF) generator and CH_4_ flow rate. The meso-plasma system was actually a modified ICP-jet plasma in combination with a planar-coiled antenna. In such a configuration, they obtained fast deposition because of a high dissociation rate of CH_4_. The CNWs deposition was performed in CH_4_/H_2_/Ar mixture. A deposition rate was increasing with increasing RF power (12–18 kW) and increasing CH_4_ flow rate (10–80 sccm), when keeping H_2_ flow constant. The highest growth rate (18 µm/min) was observed, when the flow of H_2_ was zero, what was explained by a lower etching effect of hydrogen. An increase in the plasma power and CH_4_ flow did not only change the growth rate but it also had an effect on CNWs morphology and structure. Different morphological forms including petal-like, cauliflower-like, maze-like, or floc-like structures were observed. 

## 3. A Brief Review of Patents 

As already mentioned, there is a great commercial interest in application of carbon nanowalls in different devices. In order to make this review rather complete, the most relevant patents on deposition of carbon nanowalls are listed below and briefly described.

Probably the first patent application on growth of CNWs was filed in 2007 by Hiramatsu and Hori [42]. They disclosed a method and a device for producing thin films of CNWs on solid substrates. A source gas containing carbon was introduced into a reaction chamber where plasma was sustained with a capacitively coupled generator. The authors disclose also a second radical-generating chamber which was disposed outside the reaction chamber. Hydrogen radicals were generated by decomposing radical source gas containing hydrogen using RF or another method. The hydrogen radicals were introduced into the plasma, whereby CNWs were formed on a substrate disposed on the second electrode of the CCP. The growth of the CNWs with this method was found to be rather slow (about 1 µm high-quality CNWs in approximately 5 h). The key innovative step in this patent application was application of a remote source of atomic hydrogen, which was essential for the growth of high-quality CNWs. The drawback of the method is a very long treatment time. This drawback was suppressed in the patent [43] which discloses a method and a device for deposition of carbon nanostructures where the base materials forming carbon nanostructures can be continuously fed, thus mass-production could be facilitated. The method described in [43] is actually based on the method revealed in previous patent [42] by the same group. The patent [44] further improves the method described in [42], in particular to improve the crystallinity of CNWs. However, the improvement of crystallinity had a negative effect on the growth rate, since it was reported to decrease from about 60 to 20 nm/min.

A method for growing CNWs on a solid substrate is disclosed also in the patent by Ghoanneviss et al. [45]. In this patent, a method is described which comprises mixing a predetermined amount of a hydrocarbon gas with a predetermined amount of at least one non-hydrocarbon gas, placing the solid substrate into a reaction chamber; creating gaseous radicals in the reaction chamber which comprises hydrocarbon and non-hydrocarbon radicals; applying the radicals to the solid substrate; and growing CNWs on said solid substrate exposed to said radical. This invention comprises a method where CNWs are created under atmospheric pressure. The CNWs growth with this method usually takes tens of minutes. No fluxes nor fluences of said radicals are disclosed in this patent application.

CNWs were also formed as a product in a CO_2_ reduction device with the CO_2_ reduction method disclosed in a recent patent application by Ohmae et al. [46]. This CO_2_ reduction method produces CNWs by transforming CO_2_ gas into carbon using microwave (MW) plasma chemical vapor deposition and, essentially, using water vapor as a carrier gas. In the preferred embodiment of this patent, the method based on MW plasma chemical vapor deposition is used to reduce CO_2_ gas in carbon oxide-containing gas flowing through the inside of an U-shaped reaction tube made from glass. The water vapor is used just as a carrier gas of the carbon oxide-containing gas according to Ohmae. Unlike all previously cited documents the methods disclosed in this patent application do not rely on injection of hydrocarbons into gaseous plasma. The CO_2_ gas is dissociated upon plasma conditions and CNWs are produced on a solid substrate positioned inside the glass tube. The inventors claim a CO_2_ reduction system which has the U-shaped CO_2_ reduction device whose gas exhaust tube is connected with the gas introduction tube. The inventors also claim a CO_2_ reduction method which produces CNWs by conversion of CO_2_ gas into carbon source using MW plasma CVD method and water vapor as a carrier gas. In fact, the tube is mounted into a MW waveguide of such a shape that an extremely large electromagnetic field is obtained right at a bend of the tube, therefore the power density is extremely large. Unfortunately, the authors of this patent do not report the exact value of the power density, nor the substrate temperature, but both should be large in such a configuration. The scalability of the method is questionable, though. The decomposition rate of CO_2_ increases with increasing discharge power. The electric power generated by photovoltaic power generation is used for powering the MW plasma generator in one embodiment, thus making the device highly economical. The sediment (i.e., the CNWs film) as deposited by the methods of Ohmae also contains other morphological forms of carbon. 

CNWs can be used for fuel cells, lithium ion batteries, diodes, and photovoltaic devices, etc. In another patent by Hori’s group [47] a method for manufacturing a catalyst layer for a fuel cell is disclosed. Here, CNWs are refined in order to enhance the power generation efficiency of a fuel cell by improving the contact of hydrogen molecules and oxygen molecules which take part in a reaction with a metal catalyst and an electrolyte in the fuel cell to sufficiently form a three-phase interface. 

The method that simplifies the process for manufacturing an electrode layer for fuel cells and improves the dispensability of the catalyst component and the electrolyte, whereby the generation efficiency of a fuel cell can be improved, is also revealed in yet another patent by Hori et al. [48].

The CNWs could be also used as a material for the negative electrode in a lithium battery. Tachibana and Tanaike [49] disclose the negative electrode material for a lithium ion battery. This material is prepared using as minute graphite material, flaky CNWs constituted of aggregates in which crystallites having a 10 to 30 nanometer range are highly oriented. A thin lithium battery which uses the innovative material is also provided. There are four other patents on CNWs for negative electrodes for lithium batteries [49,50,51] and a patent disclosing application of CNWs for a positive electrode [52].

CNWs can be also used as a part of a sample substrate for laser desorption ionization mass spectrometry (LDI-MS) as described in a patent [53]. CNWs are known as excellent absorbents because of their morphology, structure, and composition. Therefore, their possible application can be for a saturable absorbing element with a wide absorption band, a high light absorbance, and a high modulation depth as disclosed in [54]. CNWs can be also used in medical applications, for example when they are deposited on a substrate of an implantable medical device [55]. They are also used as a raw material for producing other materials, such as graphene nanoribbons [56,57] or metal-supported nano-graphite [58].

The patents do not provide details about the particular setups or just disclose the preferred embodiments, so it is difficult to extract the deposition parameters.

## 4. Summary of Literature Review on PECVD Deposition of CNWs

In Table 1 comparison of conditions used for deposition of CNWs by PECVD is shown. According to data in Table 1, CNWs are usually synthesized by various PECVD methods. These can be microwave plasma enhanced chemical vapor deposition (MW PECVD), capacitively coupled radio-frequency plasma enhanced chemical vapor deposition (CCP PECVD), inductively coupled radio-frequency plasma enhanced chemical vapor deposition (ICP PECVD), direct current plasma enhanced chemical vapor deposition (DC PECVD), and electron cyclotron resonance plasma enhanced chemical vapor deposition (ECR PECVD). A combination of these methods is sometimes used as well as additional biasing of the substrates. Especially in the case of RF plasmas, CCP configuration is often combined with ICP or an external H radical injection [13,16]. Deposition was usually performed at low pressures, however, there were also reports on the deposition at atmospheric pressure giving much higher deposition rates [23,59]. Another way to synthesize graphene sheets was also the application of a discharge in a liquid where the carbon precursor can be either the electrode material or the liquid medium [60,61]. A solution containing graphene sheets was then filtered to collect graphene sheets. Li et al. synthesized graphene sheets by pulsed arc discharge in water with petroleum asphalt as a carbon source [61]. Typical synthesis time was 20 min. On the contrary, Lee et al. synthesized graphene flakes by plasma generated between two carbon electrodes which were immersed in distilled water [60]. Recently, Amano et al. synthesized graphene flakes in ethanol with added iron phthalocyanine [62]. The synthesis time was only 5 min. 

As already mentioned in the introduction and also shown in Table 1, the growth rate and quality of CNWs can be controlled by increasing gas pressure or/and flow, discharge power, and substrate temperature. Especially, addition of H_2_ and Ar has an important influence on the quality of CNWs; therefore, the right proportion of gasses is needed for optimal CNWs deposition. Higher gas flow rates usually give higher growth rates, but also higher etching rates and loss of a desired morphology; therefore, flows and ratios should be optimized for particular applications of CNWs thin films.

As shown in Table 1, CNWs were successfully deposited to various substrates, electrically conductive and nonconductive. When first invented, deposition was performed with the help of the catalysts [36]. Nowadays, PECVD deposition is usually performed without any catalyst. As reported in the literature, deposition was successfully performed on materials such as Cu, GaAs, Si, SiO_2_, sapphire, Al_2_O_3_, Mo, Zr, Ti, Hf, Nb, W, Ta, stainless steel, MgO, TiN, Quartz glass, carbon paper, and even on non-flat surfaces such as carbon fibres and Ni foam. Yu et al. [63] managed to synthesize patterned CNWs. CNWs were grown on a gold pattern made of a network of squares and other geometrical structures that were coated on the SiO_2_ substrate before the deposition by plasma methods. 

A rather high temperature is required for deposition of CNWs. Temperatures reported in the literature are usually in the range of 600–800 °C for PECVD methods. Sometimes also temperatures higher than 800 °C were reported (up to about 1000 °C). In some cases, authors managed to deposit CNWs also at temperatures lower than 600 °C (see Table 1), depending on the substrate material. Temperatures required for CNWs deposition on glass (~400–500 °C) were usually lower than for metals (~600–800 °C) [28]. Nevertheless, despite high temperatures that are still needed for PECVD methods, they still enable deposition at temperatures lower than conventional thermal CVD methods. It is interesting, however, that temperatures of approximately 500 °C were reported for hot-wire CVD deposition of CNWs on a stainless steel substrate or quartz glass with Ni catalyst [64,65].

## 5. Comparison of Available Literature

The prior state-of-the-art can be summarized as follows:

- Either gaseous plasma or hot wires are used for production of reactive carbon-containing molecules that stick to the surface substrate and cause growing of CNWs on said substrate;
- Reactive carbon containing molecules are usually produced from hydrogenated carbon precursors, sometimes fluorinated, or from carbon oxide
- Precursors are essentially gaseous and are continuously leaked into a reaction chamber to facilitate growing of CNWs. The gases are continuously removed from the reaction chamber;
- Hydrogen is leaked into the reaction chamber simultaneously with hydrogenated carbon precursors in order to obtain good quality nanowalls. Noble gases are often added into the gas mixture leaked into the reaction chamber to alter the quality of CNWs
- Metallic catalysts were applied in early documents but have been omitted later;
- Elevated temperatures of the substrates (usually in the range of 600–800 °C) are needed for CNWs growth.

**Figure 4:**
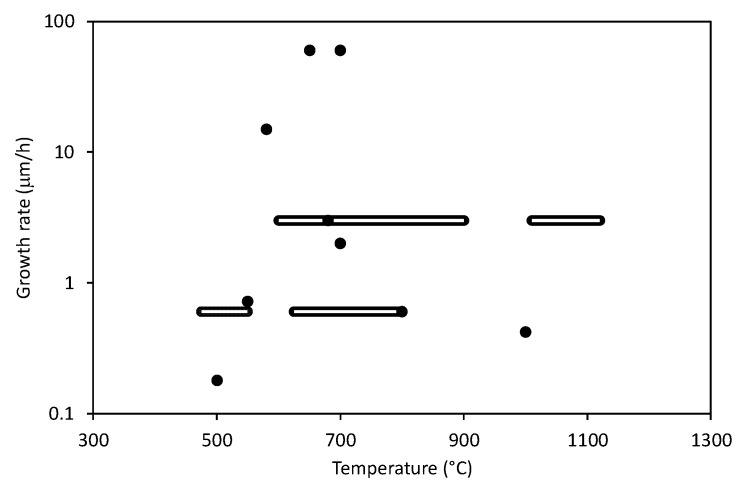
The growth rate versus the temperature as reported in literature shown in Table 1. The dots represent results in the cases when the authors performed experiments at a constant temperature. Some authors reported a range of temperatures during deposition—these results are represented with longitudinal bars.

**Figure 5:**
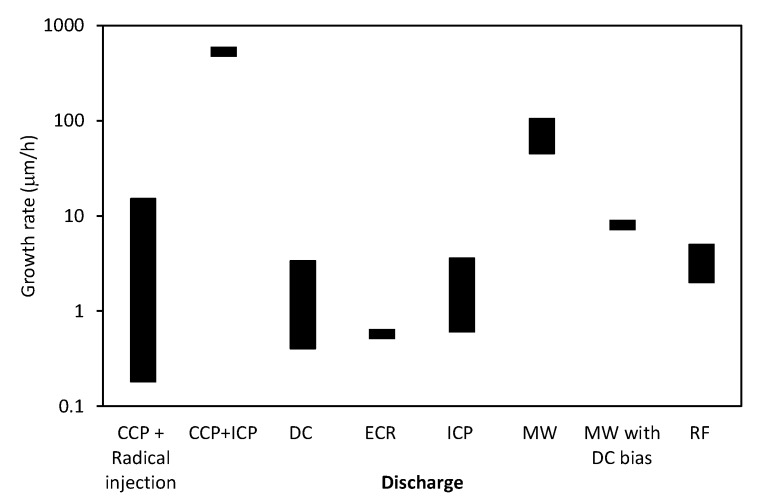
The growth rate for different types of discharges as reported in literature shown in Table 1. The height of the bars indicates the range of growth rates found in the literature.

**Figure 6:**
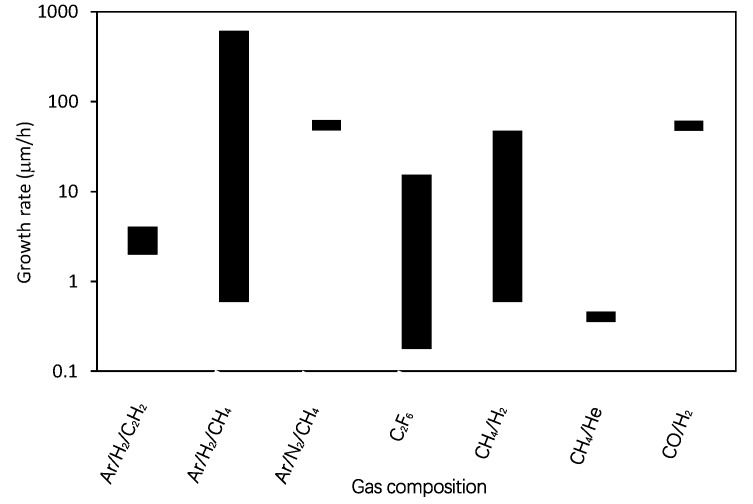
The growth rate for different gases as reported in literature shown in Table 1. The height of the bars indicates the range of growth rates found in the literature.

Different authors used different experimental setups so any comparison of results might not be scientifically perfect. Still, it is interesting to draw at least some correlations. Of particular importance is the growth rate versus the parameters reported in literature cited in Table 1. Figure 4 reveals the growth rates (where reported) versus the substrate temperature (where reported), Figure 5 the growth rates for different gas mixtures, and Figure 6 the growth rates for different discharges. As mentioned above the results summarized in Figure 4, Figure 5 and Figure 6 are based on statistical evaluation of available literature from different authors as presented in Table 1. 

Let us first examine Figure 4 which represents the growth rate of CNWs versus reported substrate temperature. The results are scattered widely, which is explained by different experimental conditions adopted by different authors. It seems that the surface temperature alone is not a decisive parameter regarding the growth rate of CNWs. Obviously, other parameters play a more significant role as long as the growth rate is the merit. There are a couple of dots in Figure 4 that stretch from others: i.e., the measured growth rate at about 60 µm/h. Both results were obtained using microwave discharge for plasma sustenance. The MW plasma adopted by Teii [30] and Mori [20] is known for the high power density, so this parameter may be more important than the substrate temperature. The power density, of course, influences the heat dissipated on the sample upon plasma treatment, and the heat in turn influences the substrate temperature. Any sample exposed to plasma is heated by bombardment with positive ions, neutralization of charged particles, recombination of radicals (in particular atoms), accommodation of any metastables, and absorption of light quanta. The prevailing mechanism depends on fluxes of reactive species and biasing. Unfortunately, only a few authors mention these parameters, so it is difficult to deduce the heating power. In any case, the fluxes usually increase with increasing power density of the discharge until the saturation is reached. For example, the atom density next to the sample surface (and thus the atom flux onto the sample) increases with the power density, but it also depends on the properties of any material facing plasma. Carbon nanowalls should represent an almost perfect sink for atoms because they are trapped in gaps between neighboring walls, therefore they experience numerous collisions with walls before being able to escape. At each collision there is a certain probability for recombination to parent molecules and because the collisions are numerous, only few atoms are able to avoid surface recombination on a material of such a rich morphology as CNWs. In fact, one of the highest recombination coefficients was recently reported by Zaplotnik et al. [69]. Unfortunately, none of the authors cited in this review reported the atom (usually hydrogen) flux on the sample surface. 

The probability for surface neutralization of positive ions is close to 100% thus the heating by this mechanism could be deduced if plasma density is measured. The ions are accelerated when crossing the sheath next to the sample surface and the kinetic energy gained is often between 10 and 20 eV (depending on the plasma potential and the ion mass); therefore, this heating mechanism is easily evaluated if the plasma density and electron temperature are known. Again, only few authors reported these parameters. The heating by ions is of course enhanced if the sample is biased, but in such a case, the thermal contact between the sample and the electrode is usually good so biasing itself does not assure for a higher sample temperature. 

To understand the influence of a discharge type on the CNWs growth rate we summarize the results reported by various authors in Figure 5. According to literature shown in Table 1 and description in this review paper, various discharges were adopted. Unfortunately, the discharge power density is almost impossible to deduce from a good number of papers. Still, the results summarized in Figure 5 are useful for giving a hint on the role of a discharge in growth of CNWs. The highest growth rate was observed when combining the CCP with ICP. Unfortunately, only one author reported such a large growth rate [41]. The ICP in the H-mode is known for its ability to absorb large RF powers in small volumes so it can be concluded that the large power density is beneficial for fast growing of the CNWs. Furthermore, the MW discharges also provide high growth rates. As mentioned above, these discharges are also capable of sustaining dense plasma in a rather small volume. The results summarized in Figure 5 therefore indicate that the large power density is highly beneficial for a rapid growth of CNWs.

Finally, it is worth discussing the results of Figure 6, in particular because several authors stressed the influence of a gas composition on the growth of CNWs. As in Figure 4 and Figure 5, the results are scattered over a couple of orders of magnitude. The high growth rate using a mixture of carbon monoxide and hydrogen can be explained by a very high power density in plasma sustained by MW discharge in a small volume, despite using a relatively low power of 60 W [20]. The results obtained using other gases or gas mixtures are scattered so much that it may be concluded the gas mixture is not the key parameter governing the growth rate of CNWs. 

## 6. Challenges and Roadmap

The binding energy of carbon atoms in a hexagonal structure is much larger than between the graphene layers so it is natural that the synthesis of wall-like structures is dominated as long as the weakly bonded atoms are removed continuously upon growth of CNWs. The removal of such “wrongly deposited” atoms is assured by using plasma species that react chemically with weakly bonded carbon atoms and most authors agree that H atoms are particularly useful. Unfortunately, the supply of H atoms onto the surface upon the growth of CNWs seems to be too small to enable immediate removal of “wrongly deposited” carbon atoms and thus high-quality CNWs so elevated temperatures are needed, because chemical etching of carbon materials by atomic hydrogen increases with increasing sample temperature. The particular morphology of CNWs depends on deposition parameters and it has been suggested by numerous authors that bombardment of the sample with positively charged ions upon CNWs deposition is beneficial for the growth of vertically oriented (as opposite to randomly oriented) graphene sheets.

The challenges in deposition of CNWs are apparent from the text in this review paper. Although numerous authors discussed the influence of various plasma species on the growth kinetics, very few reported about the fluxes or fluences of plasma species onto the substrates. The greatest immediate challenge is therefore measuring plasma parameters. The key parameters are densities of radicals next to the substrate surface and corresponding fluxes onto the surface. While current techniques for plasma diagnostics allow for measuring densities of a variety of species they have rarely been applied. An important challenge is also determination of gradients of reactive species which appear next to or within the samples due to the loss of radicals on the surface. 

A great challenge for any future application of CNWs is upscaling. Best plasma parameters are usually found in small experimental reactors of sample size measured in cm^2^. Upscaling plasma of the right parameters to large systems is always a scientific and technological challenge. To make CNWs useful on an industrial scale, upscaling to systems that enable deposition of CNWs on large surfaces, at least as large as wafers, is essential. Most currently reported deposition rates are prohibitively slow therefore other solutions should be considered. Preferred deposition of any thin films for industrial application is in a continuous mode: the substrate (preferably an infinite sheet) moves through a dense plasma sustained by a suitable discharge and the deposition rate is high enough to assure a rapid deposition at a reasonable speed of the substrate. Such a mode, however, has not been adopted even for “traditional” plasma industries such as microelectronics so it might take a long time to invent techniques for fast deposition of CNWs on continuous materials. 

## Figures and Tables

**Table 1:** Methods and conditions for CNWs deposition.

Ref.	Gas	Temperature (°C)	Growth Rate or Time	Method	Substrate Material	Important Findings
[36]	CH_4_/H_2_	650–700	-	MW PECVD with catalyst and DC bias	Cu, GaAs, Si, SiO_2_, sapphire	-
[19]	CH_4_/H_2_	600–900	~several m/h	ICP PECVD	Si, SiO_2_, Al_2_O_3_, Mo, Zr, Ti, Hf, Nb, W, Ta, Cu, stainless steel 304	The growth rate was increasing with increasing temperature and CH_4_ concentration. CNWs on all substrates showed the same general morphology.
[37]	C_2_F_6_, CH_4_, CF_4_, CHF_3_, or C_4_F_8_ with H_2_	500	~180 nm/h	CCP PECVD + ICP for H radical injection	Si	The growth rate depended on the type of gas and it was the highest for C_2_F_6_/H_2_ and the lowest for CF_4_/H_2_: C_2_F_6_/H_2_ > CHF_3_ > CH_4_ > CF_4_/H_2_. CNWs did not grow in C_4_F_8_/H_2_ gas.
[38]	CH_4_/H_2_	-	~8 m/h	MW PECVD with DC bias	SiO_2_	The height of CNWs as a function of time obeyed the square root law.
[39]	CH_4_/He	1000	~7 nm/min	DC PECVD	Si	The average size and film thickness were increasing with increasing total plasma current.
[30]	Ar/N_2_/CH_4_ Ar/N_2_/C_2_H_2_	min. 650	1 µm/min	ASTex MW PECVD	Si or silica	Addition of Ar gas reduced the deposition temperature and increased the production of C_2_ dimers.
[20]	CO/H_2_	700	1 µm/min	ASTex MW PECVD	Si	High growth rate was obtained at a relatively low MW power of 60 W.
[31]	CH_4_/H_2_	~400	up to 180 s	ECR-MW PECVD	SiO_2_, glass, Cu	Deposition temperature depended on the substrate material.
[26]	CH_4_/Ar	625–800	~10 nm/min	ECR PECVD	SiO_2_/Si	The growth rate and quality of CNWs could be enhanced by increasing the substrate temperature, decreasing the distance between the MW source and the substrate, and increasing the MW power. Below 625 °C CNWs did not grow.
[16]	C_2_F_6_/H_2_ w/o O_2_	580	~25 nm/min	Radical injection CCP PECVD	Si	O_2_ gas addition reduced the amorphicity and disorder of CNWs and assisted in nucleation of CNWs.
[40]	CH_4_/H_2_	680	1 µm/20 min	RF PECVD	Cu, Si, and Si with a film of Ni or Au	Morphology of CNWs depended on the type of a substrate
[18]	CH_4_/H_2_	-	1.5 m/2 min	MW PECVD	Cu	The size of graphene sheets depended on a flow rate. A maximum was observed at 10–30 sccm.
[35]	Ar/H_2_/C_2_H_2_	700	1 µm/30 min	RF plasma beam PECVD	SiO_2_/Si, Ti, stainless steel, Quartz, MgO, carbon paper (all substrates covered with clustered Ni catalyst)	Type of the substrate material was not critical for CNWs growth. Quality of CNWs depended on pressure and Ar flow rate. Low pressure and high carrier flow rate was found to be optimal.
[41]	Ar/H_2_/CH_4_	-	~10 µm/min	Mesoplasma (CCP+ICP) PECVD	Si	Growth rate was increasing with increasing RF power (12–18 kW) and increasing CH_4_ flow rate (10–80 sccm). Various CNWs morphologies were observed.
[27]	Ar/CH_4_	750–900	up to 10 min	CCP PECVD	Cu	The density of CNWs increased with substrate temperature, plasma power, and deposition time.
[28]	Ar/H_2_/CH_4_	475–550	~10 nm/min	ICP PECVD	glass	The size and density of CNWs increased with increasing temperature.
[17]	Ar/H_2_/C_2_H_2_	550, 650, 750	-	RF PECVD	Si, Ni/Si, Al_2_O_3_, carbon fiber	CNWs did not grow at 550 °C. Morphology of CNWs depended on temperature, pressure, and gas flow.
[59]	Ar/CH_4_	700	~300 nm/min in lateral size	Atmospheric DC PECVD	Polished stainless steel	Growth rate is much higher compared to low-pressure synthesis.
[23]	Ar/H_2_/ethanol or hexane vapor	800	100 nm/min	Atmospheric DC PECVD	Ni	Growth rate is much higher compared to low-pressure synthesis.
[24]	Ar/H_2_/ethanol vapor	700	>15 min	Atmospheric DC PECVD	Si, Cu, stainless steel	-
[63]	Ar/CH_4_ or Ar/C_2_H_2_	-	Several min	Low-pressure PECVD	SiO_2_/Si with Au pattern	CNWs were grown on a substrate with a designed pattern.
[66,67]	H_2_/CH_4_	~1000	~50–55 nm/min	DC PECVD	Glassy carbon, Si	Substrate temperature depended on the film thickness. An increase in temperature of the substrate surface resulted in an increase in the nanowall average linear size.
[22]	p-xylene	450	20 min	ICP PECVD	Si coated with TiN	Three types of carbon nanostructured were formed depending on the flow rate: fibers, free standing nanowalls, or interconnected nanowalls.
[29]	Ar/H_2_/C_2_H_2_	200–700	60 min	RF plasma beam PECVD	Si	Strong dependence of morphology on temperature: CNTs were observed at 200 °C, amorphous carbon nanoparticles in the range of 300–400 °C and CNWs at 500–700 °C.
[25]	aluminum acetyl-acetonate + Ar	350, 425, 500	50 min	ICP PECVD	Stainless steel, Ni, Al, Si	Strong influence of the bias voltage, substrate temperature, and substrate material on the morphology of CNWs. Nanorods or thorny, straight, or curled CNWs were found.
[68]	H_2_/CH_4_	600	40 min	RF PECVD	Ni foam, copper, glass	-
[2]	Ar/H_2_/CH_4_	520–550	12 nm/min	ICP PECVD	SiO_2_	Quality of CNWs increased with plasma power and temperature.
